# Monkeypox Virus: WHO's Second Public Health Emergency of International Concern Within 2 Years

**DOI:** 10.1111/1751-7915.70142

**Published:** 2025-04-07

**Authors:** Harald Brüssow

**Affiliations:** ^1^ Department of Biosystems, Laboratory of Gene Technology KU Leuven Leuven Belgium

**Keywords:** antivirals, epidemiology, monkey pox, PHEIC, Poxviridae, vaccines, zoonosis

## Abstract

An upsurge of monkeypox disease (mpox) cases with clade I virus in Central Africa led WHO to declare a Public Health Emergency of International Concern for a second time shortly after the worldwide clade II mpox epidemic in 2022/3 among homosexual men. In the Democratic Republic of Congo (DRC), the annual incidence of clade I mpox, transmitted mostly from animal sources to children, increased 20‐fold between 1980 and 2007; 60,000 mpox cases occurred between 2010 and 2023. The incidence again doubled between 2023 and 2024, showing a case fatality rate of 3.3%. A new clade Ib virus was detected in 2024 in eastern DRC where mostly adults were infected by heterosexual contact. Ib was recently introduced and showed a mutation spectrum of human‐to‐human transmission. Asymptomatic mpox infections, the release of infectious virus before symptom onset in a subgroup of cases, and superspreaders complicate containment measures during the 2022 epidemic. Isolation of cases until two consecutive negative PCR tests was recommended but necessitates cheap and rapid diagnostic tests which are in development. Sexual behavioural changes during the 2022 epidemic have contributed more to the curbing of the epidemic than vaccination. The smallpox vaccine Dryvax protected children exposed to clade I mpox in DRC in the 1980s. The attenuated third‐generation smallpox Modified Vaccinia Ankara (MVA) vaccines and derivatives showed robust protection against clade IIb mpox during the 2022/3 epidemic in various study formats. Vaccine efficacy exceeding 75% was reported after two doses. mRNA in lipid‐nanoparticle encoding surface proteins from extracellular enveloped and intracellular mature virions of monkeypox virus (MPXV) induced humoral and cellular immune responses that protected macaques against mpox disease with clade I and II viruses better than MVA. Only mixtures of monoclonal antibodies protected mice from mpox. The antiviral tecovirimat showed no efficacy in two clinical trials against clade I and II mpox.

## Graphical abstract

The figure shows examples of zoonotic diseases and their affected populations. The red arrows indicate the path of monkeypox virus infections first from a wild animal reservoir (rodents, squirrels) as a zoonosis mostly to children in central and west Africa, followed by the 2022/3 epidemic with human‐to‐human transmission in men having sex with men and in 2024 also with heterosexual transmission between humans. Figure credit: This figure is excerpted from a US GAO report GAO‐12‐55: www.gao.gov/products/GAO‐12‐55 Biosurveillance: Nonfederal Capabilities Should Be Considered in Creating a National Biosurveillance Strategy. In the public domain.

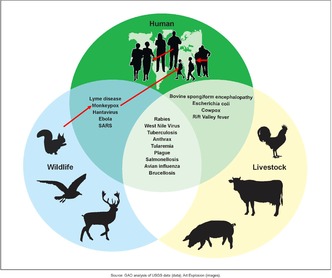

## 1 Introduction

**FIGURE 1:**
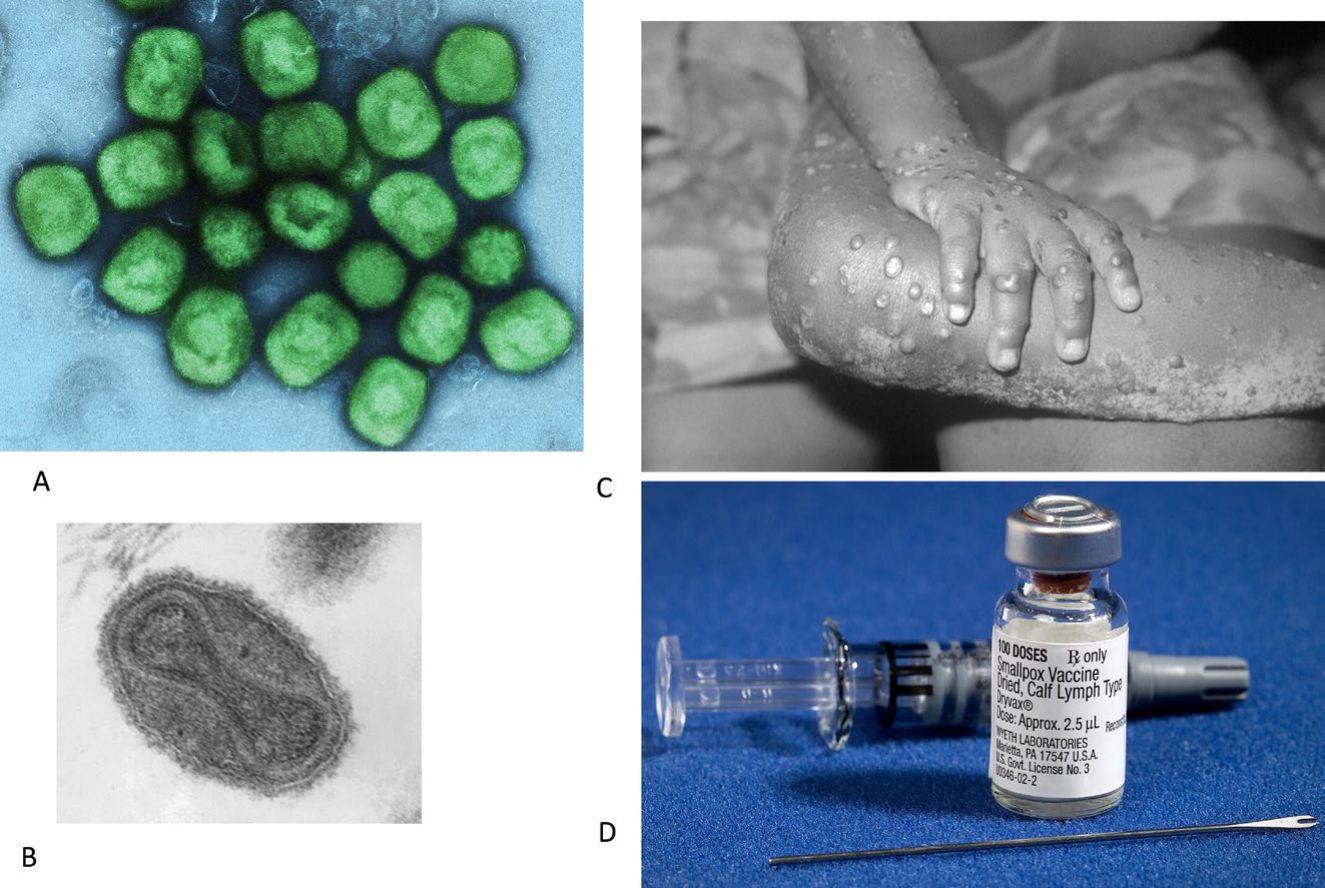
(A) Colourized transmission electron micrograph (TEM) of MPXV particles. (B) TEM showing the ‘dumbbell‐shaped’ structure inside the smallpox virion, which is the viral core, containing the viral DNA. This DNA is the source of virus replication when inside the cytoplasm of a host cell (see Figure 3). (C) Close‐up of mpox lesions on the arm and leg of a 4‐year‐old female child from Liberia. (D) Components of a smallpox vaccination kit, including the diluent, a vial of Dryvax smallpox vaccine and a bifurcated needle. Vaccinia vaccine, derived from calf lymph and currently licensed in the United States, is a lyophilized, live‐virus preparation of infectious vaccinia virus. It does not contain smallpox variola virus. Figure credits: (A) National Institute of Allergy and Infectious Diseases (NIAID), NIAID Integrated Research Facility (IRF) in Fort Detrick, Maryland. CC BY 2.0; (B) CDC/Dr. Fred Murphy; Sylvia Whitfield in the Public Health Image Library (PHIL), with identification number #1849 of Centers for Disease Control and Prevention, public domain; (C) http://phil.cdc.gov (CDC's Public Health Image Library) Media ID #2329, public domain; (D) the Centers for Disease Control and Prevention's Public Health Image Library (PHIL), with identification number #2674, public domain. All taken from Wikipedia.

The monkeypox virus (MPXV) (Figure 1A) was isolated in 1959 in Copenhagen. It belongs to the Orthopoxvirus genus of the Poxviridae family. MPXV infections were subsequently reported in monkeys from zoos and in a monkey colony maintained in a US pharma company. These observations led to the name monkeypox virus. The name is, however, a misnomer. MPXV has a broad animal host range, and the natural reservoir of this virus is squirrels and rodents from Africa. In the early 1970s, the first human monkeypox disease (mpox) cases were reported in the Democratic Republic of Congo (DRC) and in West Africa, they occurred mostly in children (Figure 1C). The viruses differed genetically, which led to the distinction of clade I (DRC) and clade II (West Africa) MPXV. The infections represented small clusters associated with bushmeat eating and were characterised by high mortality. The Orthopoxvirus genus includes veterinary pathogens such as cowpox, camelpox, horsepox and mousepox (ectromelia) viruses, Vaccinia virus (VACV) of unknown origin (buffaloes?) as well as one of the most dreaded viruses of human history, Variola virus (VARV) (Figure 1B) which caused smallpox (Brüssow 2023). To verify whether the World Health Organisation (WHO) smallpox vaccination and eradication campaign had achieved its goal, smallpox‐like skin eruptions were intensively investigated in the 1970s. No smallpox cases and only a few mpox cases were detected when screening more than 6 million children from Central and West Africa for skin lesions. In the 1980s and 1990s, sporadic cases of clade I (now subclade Ia) MPXV infections were reported in DRC, suggesting that MPXV was endemic in Central Africa. The case fatality rate (CFR) was particularly high in young children. In 2003, a small number of clade II (now subclade IIa) MPXV infections were observed in US citizens handling imported pet animals (prairie dogs) from Africa. Subsequently, MPXV infections developed a new dynamic, starting in 2017 with a clade II MPXV epidemic in Nigeria, which affected nearly 300 subjects. The patients were mostly young male adults. The situation changed again and dramatically in 2022 when a new form of clade II (now subclade IIb) West African MPXV got transmitted among males having sex with males (MSM). A worldwide epidemic largely limited to the gay population started with clade II MPXV in May 2022 and rapidly reached peak numbers with more than 30,000 monthly cases in August 2022. Most cases were observed in America and Europe. In July 2022, WHO declared mpox a Public Health Emergency of International Concern (PHEIC). After public alerts and information campaigns among MSM networks and about 100,000 mpox cases, case numbers dropped rapidly, and the PHEIC status of mpox was lifted in May 2023. Literature references and more detail for these earlier phases of the mpox epidemic can be found in a previous Lilliput review (Brüssow 2023). An upsurge of mpox cases with clade I MPXV in DRC and its spread to neighbouring countries led WHO to declare mpox again a PHEIC on 14 August 2024. In the 21st century, only Ebola has twice been declared a PHEIC. What had happened that WHO declared MPXV a PHEIC for a second time within 2 years?

## 2 The Events Leading to the Second PHEIC Alert for Mpox

In central DRC, the annual incidence of mpox increased 20‐fold between the 1980s and 2007, from 0.5 to 11 per 10,000 population, respectively (Rimoin et al. 2010). In 2007, mpox showed a clear association with ecological conditions: the annual incidence was 11, 6 and 3 cases per 10,000 population for forested, mixed and savannah areas, respectively. The average age of mpox cases was 12 years. Smallpox‐vaccinated persons had a fivefold lower risk of mpox as compared with unvaccinated persons, suggesting an 80% vaccine efficacy (VE) against mpox. The mpox incidence increase was attributed to the waning immunity of the smallpox vaccination campaign, which stopped in 1980.

The mpox situation in DRC subsequently aggravated. A large consortium led by Belgian and Congolese epidemiologists counted 60,000 clinically suspected mpox cases in DRC between 2010 and 2023 (Bangwen et al. 2025). During this time period, the annual incidence quadrupled from 2200 to 15,000 cases. Children accounted for 65% of the cases. The overall CFR was 4.6% and even higher in young children. Mpox had a focus in central DRC, but spread over time into many other DRC provinces. The disease was nearly exclusively limited to rural areas, with a dominance of rainforest over savanna areas, and it displayed a trend for yearly seasonality. When limiting the analysis to laboratory‐confirmed cases, a comparable temporal increase in mpox was observed. About 60% of the PCR tests confirmed an Orthopoxvirus infection. Within the Orthopoxvirus‐negative samples, 40% tested positive for varicella zoster virus. While this observation indicates an overestimation of mpox, this effect is more than compensated for by under‐reporting of mpox from low health care‐seeking behaviour in remote rural areas. The situation in DRC is still accelerating: when 15,000 suspected mpox cases were reported in 2023, the same number was already noted in the first half of 2024.

Imported clade I cases have been reported in Sweden and Thailand (Wang and Gao 2024). A further warning signal was a changing epidemiological situation: clusters of clade I MPXV in DRC were transmitted by sexual contact (Kibungu et al. 2024). While sexual transmission was the rule for MPXV during the 2022 worldwide epidemic, this epidemic was caused by a clade IIb MPXV. Clade I MPXV was until recently not known to be sexually transmitted. In the past, most clade I MPXV infections occurred in children from remote DRC villages. The children likely contracted the disease primarily from rodents. In September 2023, an mpox case was reported in the South Kivu region of eastern DRC, where mpox was not observed in the past. Until February 2024, a total of 241 further cases of mpox were reported in this region: 52% of the cases were young females, most of whom were sex workers. Children constituted 15% of the cases. All patients showed a cutaneous rash; 59% had fever; 42% lymphadenopathy; and two died from mpox. MPXV genome analysis revealed a new clade I virus annotated as subclade Ib. The analysis of the mutation pattern suggested human to human transmission and pointed to a recent origin of this MPXV subclade (Vakaniaki et al. 2024). The diagnostic mutation signal for human transmission was elevated TC>TT mutations (C mutating to T with an upstream T nucleotide) driven by the human apolipoprotein B mRNA‐editing enzyme, catalytic polypeptide‐like 3 (APOBEC‐3) proteins, causing cytosine deamination in viral genomes. Absence of this mutation pattern indicates lack of exposure to human APOBEC‐3 activity and therefore suggests circulation of these MPXV isolates outside of the human population, representing likely animal to human transmission events. APOBEC3 genes have expanded in primates (humans have seven genes, rodents – the likely reservoir of MPXV – only one). By causing hypermutation in the viral genome, APOBEC3 represents a host‐mediated antiviral mechanism. APOBEC3 signals have already been observed during the worldwide 2022/3 epidemic with clade IIb viruses. The basic MPXV evolutionary rate in animals corresponds to 1 nucleotide change per 3 years. Observing 42 nucleotide substitutions during the 2022/3 clade IIb mpox epidemic is thus 28 times higher than expected for circulation in an animal reservoir and likely reflected human‐to‐human transmission for clade IIb since 2016 (in Nigeria?). Mutations led during the worldwide clade IIb epidemic to sub‐lineages A.1, B.1, and A.2 (O'Toole et al. 2023).

Clinical and epidemiological traits were then explored in a prospective observational study from a hospital in South Kivu (Brosius et al. 2025). From 500 suspected mpox cases, 80% were laboratory‐confirmed MPXV infections; practically all belonged to subclade Ib. Adults accounted for the majority of cases; half of them were women, and only 20% of the patients were children. Occupation‐wise, a quarter were male mine workers, and 13% were female sex workers. Half of the adults reported transactional sex. Most patients reported contact with suspected mpox cases, either sexually between adults or via close household contacts in children. Contact of children with wild animals was rare. Fatigue, malaise and myalgia were prodromal signs before the appearance of rash. Genital rash accounted for half of the lesion counts; the median count was 40 lesions, much higher than in the 2022 clade IIb worldwide mpox epidemic. Lymphadenopathy was high in adults and fourfold lower in children. The median hospitalisation duration was 7 days; most cases were clinically mild or moderate. A third of the patients had moderate residual clinical signs 2 months after hospitalisation. However, two paediatric patients had died, and four out of six pregnant women lost the fetus due to a transplacental infection. A high rate of fetal mpox infection was also observed in a prior study from central DRC. From 4 MPXV‐infected pregnant women, only one delivered a healthy baby; two had a miscarriage in the first trimester, and one had a macerated stillborn; the fetal tissues and the placenta showed high level MPXV replication (Mbala et al. 2017). A review of the literature documented the outcome for 12 further pregnancies in MPXV‐infected mothers. Six fetal deaths were reported, and six delivered a healthy child. The outcome for three breastfed babies from MPXV‐infected lactating mothers was documented; all babies were infected, and one baby died (Sanchez Clemente et al. 2024).

Another study with 226 suspected mpox cases from South Kivu, investigated between October 2023 and February 2024, documented transmission mostly by heterosexual contact (92%). Homosexual contact accounted for only 4% (Katoto et al. 2024). Women represented 54% of the cases. These researchers noted a sequential change in mpox epidemiology over the last decades. From a zoonosis in people having wild animal contact, to limited human‐to‐human transmission in the early African phase, followed by a worldwide human‐to‐human transmission chain in MSM to heterosexual mpox transmission in Central Africa. This sequence of events reminded the changing epidemiology seen with HIV infections. They warned that war and social unrest in Eastern DRC facilitated cross border infections and that the new infection has the potential for another global spread. In 2022, war in the Kivu region induced 60,000 people from DRC to cross to Uganda. Burundi and Rwanda which reported each 2500 cases after the second WHO PHEIC declaration (Ndembi et al. 2025). Mpox cases were also, for the first time, observed in North Kivu province of DRC in a displaced person camp mediated by a close social, but non‐sexual, contact.

For the time period 2022 to October 2024, 45,600 mpox cases were observed in 12 African countries. Mortality was high with 1500 deaths indicating a CFR of 3.3%. A 2.8‐ to 4.3‐fold increase in cases was observed over this time period for laboratory‐confirmed or clinically suspected mpox cases, respectively. DRC was the most affected country. Within DRC, only four provinces provided high case numbers. No gender bias was seen, and half of the cases were in children. The most prevalent symptoms were cutaneous rash and fever. The current surge is caused mainly by clade Ib MPXV against the background of ongoing clade Ia transmission in central Africa and clade IIa in western Africa (Ndembi et al. 2025). The authors asked for possible causes explaining the surge in mpox cases. One relevant factor was the young age of the population: 85% of the DRC population is younger than 40 years and has thus not received the smallpox vaccination, which provides cross‐protection to mpox. War, conflict, displacement, and a fragile health system also contributed to the mpox surge. It is currently not clear whether the genetic differences between clade I and clade II viruses or their distinct transmission mechanisms explain the nearly 100‐fold CFR difference between them (the worldwide epidemic from 2022 showed a 0.03% CFR). It should be noted that the CFR in DRC was fivefold lower in laboratory‐confirmed than in clinically assessed mpox cases. Furthermore, a CFR of 0.3% (and not 3.3%) was reported for Burundi, Uganda, Rwanda, and Kenya, regions that were only recently affected by clade Ib, raising the question of whether epidemiological factors such as malnutrition, social upheaval, and HIV co‐infection, and not clade‐specific viral traits, have a greater effect on CFR (Ghebreyesus 2025).

A comparison with smallpox might be helpful when assessing the impact of mpox epidemics. An annual maximum of 5523 smallpox cases, leading to 710 deaths (CRF 13%), was reported for DRC in 1963 to WHO. This is in absolute terms lower than the mpox cases (38,000) and deaths (1000) reported for DRC in 2024 by clade I MPXV. However, CFR is higher for smallpox than for mpox (13% vs. 2.6%) (Ndembi et al. 2025). In contrast, by August 2024, the epidemic with clade IIb sub‐lineage B1 caused 99,176 cases and 208 fatalities in 116 countries (CFR of 0.1%) (Otieno et al. 2025). The attack rate among unvaccinated subjects living in a house with a primary mpox case was 9% compared to 4.5% in a subject living in a neighbouring house, which is much lower than comparable rates seen in DRC for smallpox, which ranged from 37% to 88% (Jezek et al. 1988).

## 3 Viral Genomics to Differentiate Mpox Epidemics

### 3.1 DRC

Intensive sequencing of MPXV genomes has been done to assess the impact of viral traits versus epidemiological cofactors on disease severity and to differentiate distinct mpox epidemics, which might occur in parallel. In this vein, the viral genomes derived from 340 mpox patients were sequenced (Kinganda‐Lusamaki et al. 2025). The patients were seeking medical help in DRC between 2018 and 2024. All sequences belonged to clade I MPXV as expected for mpox cases in central Africa. Notably, only 17 genomes from South Kivu province, collected in 2024, belonged to clade Ib and showed little diversity. The remainder belonged to clade Ia, which showed a 10‐fold higher degree of genome diversity. Five groups could be distinguished in clade Ia viruses from DRC. One was the most diverse and widespread and came from regions around the Congo river, while others originated from the central province and the southern savannah areas, respectively. Despite this geographical differentiation, co‐circulation of different clade Ia groups was observed in several areas. In some districts, up to three different viral variants were detected at the same time. No particular APOBEC3 mutation enrichment signal was seen in clade Ia viral genomes. This observation concurs with the traditional paradigm of zoonotic spillover events with limited human‐to‐human transmission. The genetic diversity between clade Ia strains from DRC suggests multiple interspecies transmission events, raising the question of the animal reservoirs from where MPXV infections entered the human population. In contrast, clade Ib viruses were restricted to recent samples of low genetic diversity from the Kivu province and showed a fivefold higher APOBEC3 mutation signal than clade Ia, supporting the interpretation of a recent introduction from a single animal source, followed by human‐to‐human transmission chains.

This conclusion concurs with two prior studies which reported genome sequences from a South Kivu hospital that defined the new clade Ib viruses (Masirika et al. 2024; Vakaniaki et al. 2024). Molecular clock analysis dated the common ancestor of the South Kivu genomes to mid‐September 2023.

When sequencing viral genomes from 11 mpox patients in the western Kinshasa province, a co‐circulation of clade Ia and clade Ib strains was detected in the summer 2024 (Wawina‐Bokalanga et al. 2024). Together with the spread of clade Ib from South to North Kivu and to adjacent eastern African countries, the detection of clade Ib in the proximity of the international city of Kinshasa represents a strong danger signal for a potential international spread of clade Ib MPXV.

### 3.2 Republic of Congo and Central African Republic

Genome sequences from 31 mpox patients of the Republic of Congo, situated to the west of DRC, collected in early 2024, revealed exclusively clade Ia viruses, separated into two subclusters (Yinda et al. 2024). One cluster resembled viruses detected in the Central African Republic (CAR), situated to the north of DRC. The other cluster corresponded to viruses currently circulating in DRC and shared sequence identity in excess of 99.5% with them (Berthet et al. 2021). As most mpox cases in CAR occurred at the northern edge of rainforests, transmissions from wild animals living in the rainforest were considered likely by the authors. Political instability increased the frequency of contact with rainforest animals in that region.

### 3.3 Nigeria

In contrast, 18 viral genomes recovered from Nigeria in 2019 all belonged to the clade IIb, more specifically to the sub‐lineage A. They were closely related to the viruses causing the 2017 outbreak in Nigeria and to exported mpox cases detected in the US, UK, Israel and Singapore. However, they were distinct from clade IIb viruses of lineage B1, which caused the worldwide mpox epidemic in 2022/3 (Ndodo et al. 2023).

### 3.4 Phylogeny Tree Analysis

Implementing measures to control mpox outbreaks will require an in‐depth understanding of how the virus is transmitted. Therefore, a survey analysing more than 10,000 MPXV genome sequences, collected from 64 countries between 1958 and 2024, presents an important basis to address this question (Otieno et al. 2025). The resolution is somewhat limited by the fact that 97% of the sequences were derived from clade IIb viruses of the B1 lineage of the 2022/3 epidemic. Clade I viruses have mostly been isolated from the Congo Basin area; the earliest sequence was from 1970. When reconstructing a temporal phylogenetic tree, the most recent common ancestor of clade I was dated to 1917. As viruses from different geographical regions often did not cluster within the phylogenetic tree, multiple introductions of clade I into local populations were deduced. The recently emerged clade Ib viruses spread beyond the Congo basin, but also clade Ia was detected elsewhere, for example in Sudan in 2005. For clade I, 96% of the sequences were derived from human isolates, with rare sequences derived from chimpanzee, shrews and squirrels. In contrast, only 12% of clade IIa sequences were from humans, while 60% were from chimpanzees, which are, however, a spillover host rather than a reservoir host for MPXV. Also, the prairie dog isolate causing the 2003 infections in the US belongs to clade IIa. The oldest clade IIa isolate dates from 1958. Clade IIa was not any longer isolated after 2018. Clade IIb was first detected in Nigeria in 2017 and continued to circulate in West Africa by human‐to‐human transmission. It was then exported to other countries in Europe, Asia, North America and North Africa, with sustained human transmission in the Eastern Mediterranean. From this clade, now called clade IIb lineage A, a descendent clade IIb lineage B.1 emerged in 2022 that caused the worldwide mpox outbreak. Clade IIb viruses exhibit a higher substitution rate than other Orthopoxviruses and display a clear APOBEC‐3 mutation signal. Clade IIa shows similar mutational processes as clade Ia viruses, lacked the APOBEC‐3 signal which suggests that they circulated outside of the human population. MPXV has a mutation rate that is 10‐fold higher than that of VARV, the agent of smallpox, and approaches that of some RNA viruses (Paredes et al. 2024). Chinese researchers investigated the spectrum of mutations in MPXV during the 2023 outbreak in Shenzhen. They observed missense (48%), synonymous (40%) and mutations in non‐coding regions (12%). Viral proteins involved in host modulation, surface exposure, DNA replication and viral assembly were affected by mutations (Zhang et al. 2024). Spanish researchers noted that variation in short tandem repeats within the low‐complexity regions of the MPXV genomes was greater than that of single‐nucleotide polymorphisms and could affect the expression of several viral proteins (Monzón et al. 2024).

## 4 Transmission Characteristics and Nonpharmaceutical Interventions

### 4.1 Phylogeographic Approaches

While the epidemiology of the current clade Ib mpox epidemic cannot be directly compared to that of clade IIb.B1 of the worldwide epidemic, the wealth of sequence information for the latter is a valuable source for epidemiological insights into possible transmission mechanisms and therefore provides hints for efficient containment measures. A phylogeographic approach with more than 1000 IIb.B1 sequences allowed several conclusions in that respect (Paredes et al. 2024). The most recent common ancestor for the worldwide epidemic was traced to West Europe in March 2022. A rapid early spread in Western Europe led to a high number of introductions into other global regions with more than 40 introduction events. There was strong evidence for viral circulation before detection of the epidemic in each global region.

### 4.2 Asymptomatic Infections

Asymptomatic MPXV infections further complicated the epidemiological situation. In studies from DRC during the 1980s, a ratio of symptomatic to asymptomatic infection of 4:1 was seen in unvaccinated contacts of clade I mpox patients (Jezek et al. 1986). Asymptomatic infections were also observed in the clade II mpox epidemic. Among 224 men attending a Belgian sexual health clinic in May 2022, four MPXV positive cases were detected; three had no mpox symptoms while being positive for MPXV in PCR tests. They yielded a replication‐competent MPXV of clade IIb.B1 and seroconverted to MPXV (De Baetselier et al. 2022). The authors concluded that testing and quarantining of individuals reporting symptoms may not suffice to contain an outbreak. This conclusion was confirmed by a study in 113 gay or bisexual men conducted between August and October 2022 in Spain, mostly migrants from Latin America. Seven were MPXV positive in PCR tests, six showed no mpox symptoms, but three shed infectious virus (Agustí et al. 2023). Also, serological surveys conducted during the 2022 mpox epidemic suggested a substantial number of asymptomatic MPXV infections. Among 400 subjects from New York, 60% of them were MSM, 6% showed Orthopoxvirus‐specific antibodies but reported no recent mpox symptoms and had not received smallpox or mpox vaccination (Pathela et al. 2024). Similarly, among 225 patients from sexual disease clinics in San Francisco who had no prior poxvirus vaccination nor received an mpox diagnosis, 8% showed Orthopoxvirus‐specific IgG during the 2022 mpox epidemic (Minhaj et al. 2023).

### 4.3 R Values and Superspreading

When analysing transmission chains resulting from MPXV introduction events, Paredes et al. (2024) identified a bimodal pattern. Only a small number of introductions resulted in a sustained expansion of local transmissions where some individuals tended to contribute disproportionately to infection events, while two thirds contributed no new infections. Clusters of identical sequences ranged from 1 to 120, the latter pointing to superspreading events. The authors of this study computed *R*_t_, the time‐varying effective reproductive number of the virus, to be between 1.5 and 3 in the initial phase of the 2022 epidemic. Already in September 2022, *R*_t_ had dropped to < 1. Notably, *R*_t_ fell below 1 before 10% of the high‐risk US population developed immunity to MPXV by vaccination. The researchers concluded that rapid pathogen detection and concomitant behavioural change were likely sufficient to curb the 2022 epidemic spread.

### 4.4 Kinetics of Viral Release

Control measures also depend on the kinetics and the amount of virus released by mpox patients and the persistence of the virus in the environment. During the 2022 mpox epidemic, Chinese researchers analysed viral positivity by qRT‐PCR in body and environmental samples from 139 mpox patients over a 3‐week period after disease onset (Yang et al. 2024). Viral load was highest in skin lesions, peaked in the first disease week, but remained high into the third week. The next highest viral loads were detected in rectal samples while saliva samples were low and close to a viral load considered unlikely to transmit the disease. Environmental samples taken around the mpox patients showed a considerable viral contamination that persisted over the observation period, particularly on the floor, bedside, and in air conditioning outlets. An MPXV‐specific antibody response developed in the first 2 weeks after symptom onset. In another Chinese study, 39 mpox patients from the 2022 epidemic were analysed over a 3‐week period after hospitalisation. Highest viral loads were again detected in skin lesions, followed by saliva samples. At discharge from hospital, 70% of the saliva samples and 85% of dry scabs were still virus‐positive; 23% of the dry scabs yielded an infectious virus upon culture. Neutralising serum antibody titers increased over the hospitalisation period, and the titers were lower in mpox patients coinfected with HIV (Guo et al. 2024). A study from the UK which followed 11 clade II mpox patients for longer than 3 months by both PCR and virus cultivation reported a median time of 12 days after symptom onset for infectious virus isolation. However, in HIV co‐infected patients, infectious MPXV was isolated for up to 103 days. The median C_t_ value in PCR for which MPXV could still be isolated was 31 (Callaby et al. 2025).

### 4.5 Serial Intervals

Dutch researchers analysed 109 paired infector‐infectee mpox cases during the 2022 epidemic. A total of 34 infectees reported a single potential infector. From these pairs, a serial interval (the time between symptom onset of primary and secondary cases) of 10 days was deduced. Presymptomatic transmission may have occurred in 5 of 18 pairs. The scientists deduced that transmission can occur from 4 days before to 8 days after symptom onset of the infector (Miura et al. 2024). US researchers calculated a serial interval of 8.5 days and an incubation period of 5.6 days (Madewell et al. 2023). The Dutch scientists estimated a reproduction number R of 1.3 to 1.6, using the average doubling time of 11–20 days during June 2022 and suggested that a suppression of 38% of secondary infections should be sufficient to push R below 1 (Miura et al. 2024).

### 4.6 Reinfection

Rhesus macaques were experimentally infected with mpox. Irrespective of whether infected intravenously, intradermally, or intrarectally, the animals developed both humoral antibody and cellular T cell immunity after demonstrating skin lesions and a marked viremia. One month later, when skin lesions had resolved, the macaques were again challenged intravenously with clade IIb MPXV. No renewed skin lesions were observed, and only a small transient viremia was seen. The marked upregulation of innate immune cell signatures (cytokine, chemokine and interferon) seen after primary infection was not observed after re‐challenge. Upon re‐challenge, the animals showed, however, a rapid activation of an anamnestic T and B cell response (Aid et al. 2023).

Based on the SHARE‐Net international clinical network documenting mpox cases from the worldwide mpox epidemic, physicians identified reinfection in eight gay patients about 100 days after the first infection. Symptom scores decreased from the first to the second infection, and the skin lesions resolved earlier in the second than in the first infection. This international consortium also identified breakthrough infections in 30 gay subjects about 200 days after they had received MVA‐BN vaccination. Breakthrough infections were characterised by few lesions (Hazra et al. 2024).

### 4.7 Nonpharmaceutical Interventions (NPI)

NPI should thus have a realistic target as demonstrated by the rapid decline of the worldwide mpox epidemic after the summer 2022 before vaccination could have an impact on transmission. One important element was information on safer sex given to opinion leaders that was spread within well‐connected networks of MSM circles. Another classical NPI is case isolation. Current guidelines for mpox suggest quarantine of infected individuals for about 3 weeks. A modelling study analysed three types of rules for ending the isolation of patients with mpox. Under a symptom‐based rule, patients remain isolated until resolution of their skin lesions, which occurs on average after 25 days. The researchers estimated that 9% of the patients might, under this strategy, still be infectious. Under a fixed‐duration rule of 3 weeks isolation, 5% of patients might still be infectious, but many individuals will be unnecessarily isolated. A solution to this dilemma is a testing‐based strategy with daily testing and release from isolation after two consecutive negative PCR tests (Jeong et al. 2024). Such an approach necessitates rapid, reliable, and cheap diagnostic tests. Formats have been designed but not yet industrially developed that allowed MPXV DNA detection in clinical samples with a 2 min lysis protocol followed by a 10 min single‐step recombinase polymerase amplification (RPA)‐CRISPR/Cas13a reaction in a vest‐pocket analysis device, suitable for a point‐of‐care setting (Wang et al. 2024).

### 4.8 Behavioural Change

Behavioural changes in the MSM network might have contributed to the rapid decline of the worldwide clade IIb mpox epidemic. To assess the potential role of these changes in sexual interaction, researchers conducted in May 2023 a survey among about 17,000 MSM subjects from 13 European and American countries. Adaptation to their sexual behaviour was reported by half of the surveyed subjects. People in this subgroup reduced their number of sexual partners (93%), avoided group sex (88%), sex‐on‐premises venues (85%) and chemsex (54%). Subjects reporting concerns about contracting mpox were twofold more likely to adapt their sexual behaviour. Subjects with vaccination (21% mainly from Western Europe and North America had received two doses) or those having experienced mpox (6%) were less likely to adapt their sexual behaviour (Prochazka et al. 2024). Mathematical modelling with data from the 2022 epidemic in the Italian MSM community indicated that the significant behavioural changes in the community led to a rapid reduction of the reproduction number, *R* value, from 1.5 to 0.6, sufficient to stop the epidemic. The model predicted that the depletion of susceptible individuals (by infection or vaccination) was not the primary driver of the decline of the epidemic. Contact tracing might have prevented a doubling of cases during the initial phase of the epidemic. In contrast, ring tracing (tracing contacts of contacts) and ring vaccination was unlikely to stop the epidemic. However, vaccinating subjects with more than 10 sexual partners per year (10% of the Italian MSM population) could reduce the *R* value to near 1 and prevent a resurgence of mpox (Guzzetta et al. 2024).

## 5 Pharmaceutical Interventions: Active Immunisation

### 5.1 Classical Smallpox Vaccines

Since 1840, smallpox vaccines were propagated in cattle; by 1880, the leading smallpox vaccine source was calf lymph (Smallpox vaccine—Wikipedia). The first generation of modern smallpox vaccines was live, unattenuated VACV, grown in the skin of cows and sheep. A freeze‐dried Dryvax vaccine was developed in the 1950s, which allowed maintenance of vaccines without refrigeration. It was administered with a bifurcated needle to the skin (scarification) leaving a vaccination scar which served as evidence for a vaccine ‘take’ (Figure 1D). However, a third of the vaccinees developed side effects including, in rare cases, encephalitis and even deaths. The second generation of VACV vaccines was propagated on the chorioallantoic membrane of embryonated eggs or in cell culture (e.g., Lister strain by Bavarian Nordic or Sanofi Pasteur). When tested in 45,000 children from Indonesia, they had the same efficacy as the calf lymph vaccine but also the same side effects. ACAM2000 was developed from viral clones of the Dryvax vaccine and was propagated in cell culture for mass production and is used as smallpox vaccine stockpiles in the US. The FDA approved it in August 2024 also for the prevention of mpox. The Modified Vaccinia Ankara (MVA) strain is a third‐generation smallpox vaccine. A VACV strain from Turkey underwent in Munich more than 500 passages on chorioallantoic egg membranes, which resulted in the loss of 14% of the viral genome. MVA cannot replicate in human cells. It was used in West Germany in the final phase of the smallpox eradication campaign. MVA‐BN (for Bavarian Nordic as producer) was also propagated in cell culture (likewise known under the name Jynneos). It is administered by subcutaneous or intradermal injection. It is safer than ACAM2000 and of comparable immunogenicity and has also been approved for use against mpox. Another third‐generation vaccine is LC16m8 (for Lister clone 16, medium pocks, clone 8) developed in Japan by growth in cell culture and on chorioallantoic membranes. It contains the complete vaccinia genome except for a truncated viral membrane protein B5. It was approved in Japan after testing in 50,000 children.

### 5.2 Early Observations

Work conducted between 1980 and 1984 explored the efficacy of smallpox vaccination on the prevention of mpox in DRC. Overall, 2510 contacts of mpox patients were evaluated for the development of mpox. Secondary mpox was seen in 10% of household contacts without vaccination scars compared to 1.5% in contacts with vaccination scars from prior Dryvax vaccination, indicating the protective efficacy of this smallpox vaccine also against mpox. Dryvax vaccination protected also against asymptomatic MPXV infections (Jezek et al. 1986).

### 5.3 Modern Vaccination Trials With Classical VACV

In macaques immunised with either Dryvax or MVA and subsequently challenged with MPXV, both vaccines protected the animals against mpox disease symptoms, but Dryvax suppressed viremia better than MVA. However, when immunisation and challenge were shortened to a 4‐day interval, MVA showed superior protection over Dryvax, indicating a quicker onset of immune protection (Earl et al. 2008). MVA‐BN vaccine was also given to 87 children exposed to mpox cases in England as a post‐exposure prophylaxis (Ladhani et al. 2023). None developed serious adverse effects or mpox disease. All children developed antibodies against MPXV antigens B6 and B2, and a robust T cell response to MVA‐BN virus.

British researchers evaluated MVA‐BN vaccine effectiveness against laboratory‐confirmed symptomatic mpox in an MSM cohort using a case‐coverage approach whereby vaccination rates among 363 cases were compared with population vaccination coverage. A single dose showed a vaccine efficacy (VE) of 78% against mpox during the 2022 epidemic in the UK. However, this efficacy was only achieved 14 days after immunisation; at earlier dates, no vaccine protection was observed (Bertran et al. 2023).

Similar data were described for Spain in a national retrospective cohort study (Fontán‐Vela et al. 2024). During the 2022 mpox epidemic, MVA‐BN pre‐exposure immunisation was offered to subjects eligible to receive pre‐exposure prophylaxis for HIV infection. Each day, individuals receiving a first dose of the vaccine were matched to an unvaccinated subject, resulting in 5660 pairs. No protective effect of MVA‐BN was seen during the first week after immunisation, while a VE of 65% and 79% was observed after 1 and 2 weeks of immunisation, respectively. A case–control study was conducted in the US between August 2022 and March 2023 with 300 case patients and 600 age‐ and region‐matched controls; all subjects were MSM. VE against mpox was 75% for one dose and 86% for two vaccine doses; no difference was seen between subcutaneous and intradermal administration of the vaccine (Dalton et al. 2023). In September 2022, about 200,000 US citizens at risk of mpox had received two doses of the JYNNEOS vaccine (identical with MVA‐BN). Subsequently, 9500 mpox cases in the MSM group younger than 50 years were analysed by vaccination status. Mpox incidence among unvaccinated persons was 9.6 and 7.4 times as high as that among persons who had received 2 and 1 JYNNEOS vaccine doses, respectively. Only mpox cases occurring 2 weeks after immunisation were counted (Payne et al. 2022).

In another US study, 2200 mpox case patients were matched to 8300 control patients. Overall, 25 case and 355 control patients had received a full course with two doses of JYNNEOS (MVA‐BN) vaccine, indicating a VE of 66% in this observational study. For subjects receiving only one vaccine dose, VE was only 36%. The vaccine was applied subcutaneously or intradermally. Notably, intradermal injection needs only a fifth of the subcutaneous dose (Deputy et al. 2023). Another way of applying limited vaccine to a large high‐risk population is to use only one dose instead of the recommended two doses. This strategy was tested in Israel with an observational retrospective study. About 2000 at‐risk subjects were identified in a health register; half of them were vaccinated with one dose of JYNNEOS (MVA‐BN). Overall, five of the vaccinated and 16 of the unvaccinated subjects experienced mpox after vaccination. VE was with 86% high, but the low number of mpox cases limits the study conclusion (Wolff Sagy et al. 2023).

A meta‐analysis of 33 vaccine studies conducted mostly with MVA‐BN in gay and bisexual men during the 2022 worldwide epidemic indicated a VE of 76% for one and 82% for two vaccine doses. Post‐exposure VE was only 20%, but more data are needed as well as data for non‐MSM populations and clade I virus (Pischel et al. 2024) Another meta‐analysis of vaccination trials with MVA‐BN reporting VE against mpox and immunogenicity to VACV found a significant correlation between VE and VACV‐binding ELISA antibody titers. A second dose increased the antibody titre tenfold over a one‐dose regime, but increased VE only moderately. Delaying the time between the first and second dose increased the VACV‐specific antibody titre and prolonged the durability of protection (Berry et al. 2024).

### 5.4 mRNA Vaccines

**FIGURE 2:**
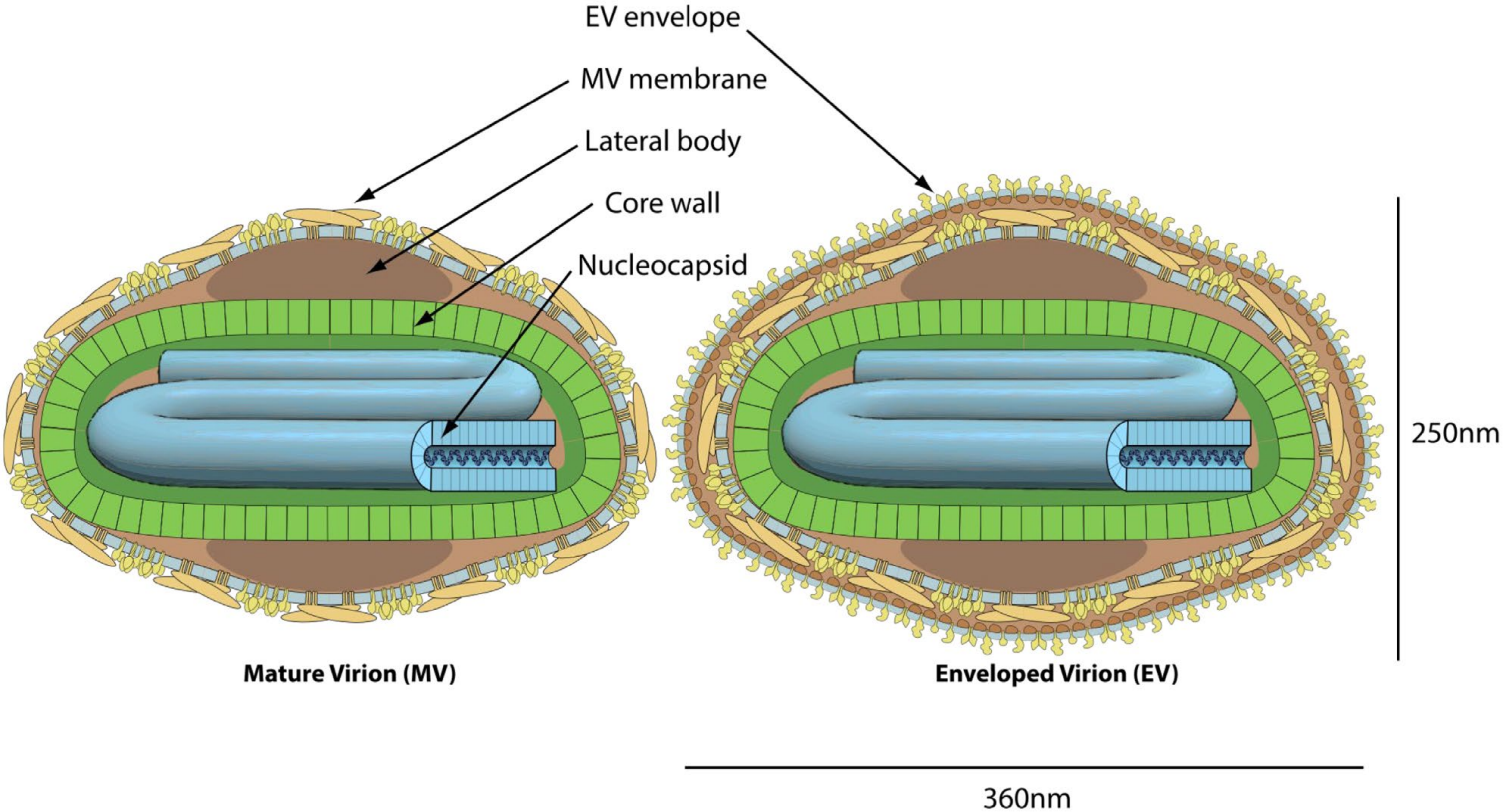
Schematic drawing of the structure from an intracellular mature virion (MV) (left) and an extracellular enveloped virion (EV) (right) of a member of the poxvirus family. Figure credit: ViralZone, SIB Swiss Institute of Bioinformatics: https://viralzone.expasy.orG CC BY 4.0. Taken from Wikipedia.

Efficient VACV‐based vaccines were thus available that also protected against mpox and stockpiles were maintained. However, these stocks were insufficient when meeting the needs of the worldwide mpox epidemic. Therefore, the pioneers of the mRNA vaccine producers against SARS‐CoV‐2 extended this platform to MPXV (Zuiani et al. 2024). They developed nucleoside‐modified mRNA encoding the clade IIb MPXV antigens A35 and B6 (surface proteins from infectious extracellular enveloped virions, EV), as well as M1 and H3 (surface proteins from infectious intracellular mature virions, MV) (Figure 2). In mice, twice immunised with each mRNA species individually or combined into a trivalent or quadrivalent mRNA vaccine, all mRNA vaccines induced a robust humoral and cellular immune response against the targeted antigen. The multivalent and the monovalent M1 mRNA vaccines also induced robust neutralising antibody (Nab) titers against MPXV and VACV. Multivalent, but not A35 + B6 vaccines protected mice against challenge with clade IIb and clade I MPXV. Multivalent and monovalent (except H3) mRNA vaccines protected mice also against VACV challenge. Multivalent mRNA vaccines given in two 30 μg doses assured survival in macaques challenged by the intratracheal route with clade I MPXV. The mRNA vaccine prevented lesion development and attenuated, but did not prevent viremia and weight loss. Clinical evaluation of BNT166 is underway with a phase I/II clinical trial (NCT05988203).

Chinese researchers had chosen three MV proteins (M1, E8, A29) and two EV proteins (A35, B6) for a pentavalent mRNA vaccine. Two intramuscular injections with 200 μg RNA induced IgG ELISA antibody increases against all five proteins and a 100‐fold and 50‐fold Nab increase to VACV and MPXV, respectively. Significant antigen‐specific CD4^+^, but no CD8^+^ T cell responses were measured in vaccinated macaques. Upon challenge of the monkeys with a circulating clade IIb MPXV from China, the vaccinated animals showed decreased mpox skin lesions, suppressed viremia and no virus excretion, and reduced cytokine production when compared to control macaques. The researchers also explored the immune response to the pentavalent mRNA vaccine in naïve and immunodeficient rhesus monkeys. Immunodeficiency was induced by infection with simian immunodeficiency virus (SIV). A robust antibody and a somewhat reduced CD4^+^ T cell response against the MPXV antigens were observed in the SIV‐infected compared to control rhesus monkeys (Ye et al. 2024).

Macaques were twice immunised with a mRNA lipid‐nanoparticle vaccine containing four MPXV mRNAs (MV: A29, M1; EV: B6, A35, 150 μg), with MVA (10^8^ pfu) or with buffer as control. One month after the boost vaccination, the macaques were intravenously challenged with a lethal dose of clade I MPXV. Both mRNA and MVA vaccinated macaques survived while 80% of the control animals died. The mRNA vaccine also protected against morbidity while MVA immunised macaques developed severe and grave disease which was, however, attenuated compared to control animals. Morbidity was quantified by lesion counts, viral burden in blood and in the throat, weight development and disease duration. With all criteria, mRNA achieved superior protection over MVA vaccination. The mRNA vaccine induced a higher clade I and II MPXV Nab titre than MVA immunisation. The mRNA vaccine also induced better Fc‐functional activities (complement binding, phagocytosis, and NK cell induction) than MVA. Cellular immune responses were not measured in this study. As the mRNA vaccine also reduced the viral titre in throat swabs, one might even expect a vaccination effect on viral transmission and not only on disease prevention. A clinical trial is underway (NCT05995275) (Mucker et al. 2024).

Another study in rhesus monkeys compared the efficacy of MVA, ACAM2000 or Ad35 vector–based vaccines, expressing L1/B5 or L1/B5/A27/A33 MPXV proteins, against an intravenous challenge with a high dose of clade IIb MPXV. All vaccines provided protection, but to various degrees. ACAM2000 mediated complete protection, while MVA and the adenovirus‐vectored vaccine only conferred incomplete protection. Protection correlated with Nab titers (Jacob‐Dolan et al. 2024).

## 6 Passive Immunisation

A report from 2005 demonstrated that macaques which received an immunoglobulin preparation from recently vaccinated subjects were protected from lethal challenge with MPXV, but they still developed skin lesions, increasing in number with decreasing Nab titers. No protection was achieved in this animal model with normal immunoglobulin preparations (Edghill‐Smith et al. 2005). Another group developed monoclonal antibodies (mab) against the VACV B5 (corresponding to B6 in MPXV) antigen, which displayed VACV neutralising activity and protected mice against a lethal challenge with VACV, but viral load was only moderately decreased compared to controls (Zhao et al. 2024). US researchers developed 89 mabs from immune human subjects. Half of them displayed in vitro neutralising activity and many were cross‐reactive against several Orthopoxviruses, including MPXV. However, most individual mabs reduced viral plaque numbers by only 70%. It needed a mixture of six mabs (directed against MV proteins D8, A27, H3, and L1 and EV proteins B5 and A33) to achieve a good in vivo protective activity in mice challenged intranasally with a lethal dose of VACV. Prophylactic application of this mab mixture achieved a 10^6^‐fold reduction of the virus load in lungs, and all treated mice survived. In immunodeficient mice, the mab mixture achieved a sterilising immunity and assured a 100% survival (Gilchuk et al. 2016). A human subject vaccinated against smallpox yielded two mabs, which bound distinct epitopes on MPXV B6 protein. Upon intraperitoneal injection, these two mabs protected VACV‐challenged mice against weight loss and modestly reduced lung titers by tenfold compared to controls (Zhao et al. 2024). A combination of two mabs directed against protein D8 and A33 found on MV and EV, respectively, protected mice from MPXV‐induced mortality and morbidity when given up to 3 days after viral challenge (Tamir et al. 2024).

## 7 Antivirals

**FIGURE 3:**
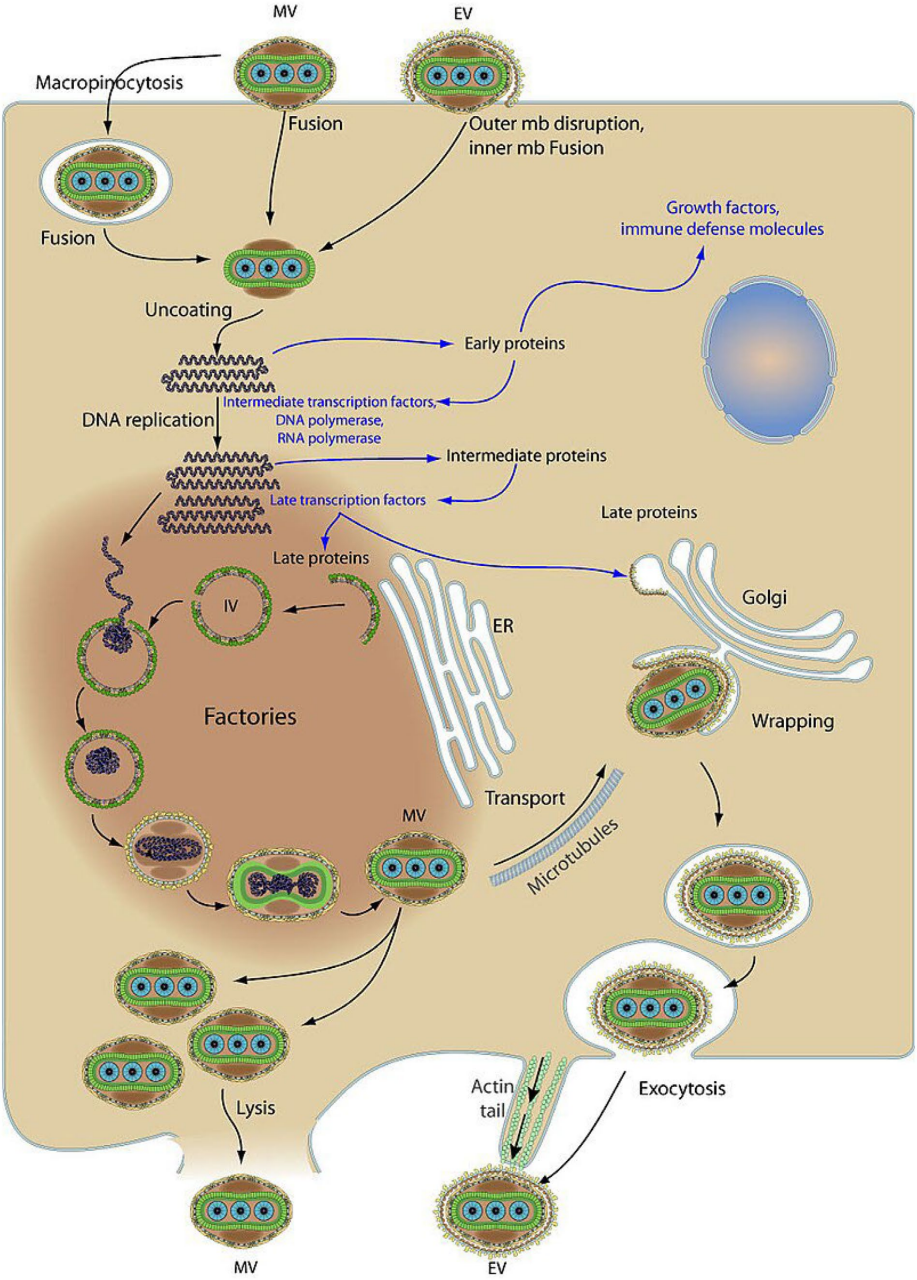
*Poxviridae* replication cycle. Figure credit: ViralZone, SIB Swiss Institute of Bioinformatics, see https://viralzone.expasy.org/, CCBY‐SA4.0. Taken from Wikipedia.

Tecovirimat binds the peripheral membrane protein F13 of EV, which is conserved across Orthopoxviruses. F13 elicits production of wrapped virions, an intermediate step of intracellular virus maturation (Figure 3). The antivirals cidofovir and brincidofovir target the DNA polymerase of Orthopoxviruses. These three drugs inhibited diverse MPXV isolates from the 2022 epidemic in cell culture at concentrations that were achieved after oral dosing in humans (Bojkova et al. 2023). In vitro inhibition was observed for clade Ia, Ib, IIa and IIb MPXVs. A few tecovirimat‐resistant MPXV mutants have been described in immunocompromised mpox patients that were treated for extended periods. However, these mutants were not seen in clade Ib isolates and only with low frequency in clade IIb isolates (Postal et al. 2025).

Macaques infected intravenously with a lethal dose of MPXV and treated at Days 4 or 5 (when pock lesions appeared) with tecovirimat showed 100% survival when treated with 3 mg drug/kg body weight. With further treatment delay to Day 6 after viral inoculation, the survival rate dropped to 50%. Five daily tecovirimat doses were associated with higher survival than three daily doses. The trial was conducted under FDA's Animal Efficacy Rule for the treatment of smallpox. In a safety evaluation with 452 human subjects treated twice daily for 14 days with 600 mg tecovirimat, adverse events were observed with similar frequency as in the placebo group (Grosenbach et al. 2018). Tecovirimat was approved by FDA to treat smallpox and can be used for mpox under an Expanded‐Access Investigational New Drug (EA‐IND) protocol. In a US study, 13 patients with advanced HIV who experienced severe mpox were treated with an extended tecovirimat course. Despite treatment, they experienced prolonged hospitalisation and high mortality. Notably, half of the patients showed a viral mutation that may indicate tecovirimat resistance (Garcia et al. 2024).

In the US, tecovirimat was prescribed under EA‐IND for over 7100 patients with painful anogenital lesions during the 2022/3 mpox epidemic. For 1600 patients an outcome was documented, but since they were not part of a clinical trial, neither safety nor efficacy could be demonstrated (Yu et al. 2024). Two recent controlled trials were conducted with tecovirimat. The STOMP trial, which enrolled MSM patients from four continents with clade II MPXV infections during the worldwide mpox epidemic, was stopped for futility after 75% of the targeted patients were enrolled, and no treatment effect could be documented. The PALM007 trial enrolled 600 children and adults with clade I mpox disease from DRC. Tecovirimat treatment had no effect on time to healing of skin lesions, virus levels in blood, skin lesions and importantly, no effect on mortality, which was 1.7% in both the treatment and placebo groups (Cohen 2025).

Not much evidence exists for the efficacy of other drugs against MPXV infection. Brincidofovir showed a modest survival effect in prairie dogs intranasally challenged with a lethal dose of MPXV. When the drug was given before or together with the challenge virus, half of the animals survived; when given 1 day after infection, only 25% of the animals survived compared with 10% in the placebo group (Hutson et al. 2021). Otherwise, two cases of severe disseminated mpox infection in renal transplant recipients were successfully treated with brincidofovir (Alameer et al. 2024).

Antiviral research against MPXV clearly needs new compounds targeting distinct viral proteins. German researchers conducted a multi‐omics analysis of the transcriptome, proteome, and phosphor‐proteome signatures of MPXV‐infected primary human fibroblasts to identify new targets. They identified perturbations of immune‐related pathways and changes in the dynamic phosphorylation of both host and viral proteins. These infection‐elicited molecular fingerprints identified nearly 700 drug targets. Based on these insights, they selected 52 drugs. As a proof‐of‐concept drug target validation screen, they tested these drugs in cell culture for an attenuated MPXV cytopathic effect, for growth inhibition of a VACV reporter and for reduction in MPXV mRNA accumulation. They identified two candidate antiviral compounds. Interestingly, tecovirimat, while reducing the amount of released virus, did not inhibit intracellular viral mRNA accumulation or cytopathic effects (Huang et al. 2024).

## 8 Political Considerations

The WHO's Contingency Fund for Emergencies released US$ 1.45 million for fighting mpox, with more to come with the PHEIC declaration. Commentators in a leading medical journal judged these funding levels insufficient to support a robust emergency response (Gostin et al. 2024). The mpox Continental Preparedness and Response Plan for Africa, co‐led by WHO and Africa CDC, formulated 10 pillars of action. It pledged financial resources but for a timely implementation an increased African leadership is pivotal for success (Abubakar et al. 2024). Likewise, when the WHO declared a second PHEIC for mpox, Japan has pledged up to 3.6 million doses of its LC16m8 vaccine, and the European Union agreed to distribute 200,000 doses while the US offered just 50,000 vaccine doses. However, even when taking Japan's pledges at face value, this still falls short of the demand for 10 million vaccine doses expressed by the Africa CDC. When resources are limited, decisions by local public health and political authorities are needed. Model calculations showed that vaccinating 80% of all children younger than 5 years in endemic regions such as DRC could lead to a 27% reduction in cases and a 43% reduction in deaths, but still require 10 million vaccine doses (Savinkina et al. 2024). Some lessons from the COVID‐19 pandemic have apparently not been learned. For example, African countries still lack the resources to track the disease, the facilities to make their own vaccines, and a regulatory infrastructure in the form of an African Medicines Agency (Anonymous Nature 2024). PHEICs are by definition a global threat to health that needs a coordinated response from leaders everywhere. A response includes the training of health workers, the organisation of genomic and epidemiological surveillance, conducting clinical trials, the development of diagnostic tools, and monitoring of animal reservoirs (Anonymous Lancet 2024). Much fewer viral genome sequences have been determined for clade I MPXV from Africa than for clade II in the northern hemisphere, which hampers the delineation of viral variants and the design of containment measures, which are most efficient when implemented within 100 days after an outbreak (Wang and Gao 2024).

After a pandemic such as COVID‐19, people are all too eager to forget their painful experiences. Psychologically, it might be understandable to suppress past pandemic experiences to maintain an optimistic view into the future. Rationally, this is not a helpful attitude. Not learning the lessons from a past painful experience and taking appropriate actions accordingly means risking living again the same painful experiences. Denying scientific evidence is self‐harming. The COVID‐19 pandemic has revealed not only a loss of rationality in substantial parts of our societies but also a flood of health misinformation and deliberate disinformation. As The Lancet wrote, health misinformation was weaponised as propaganda, exploiting fear, undermining public trust, and hindering collective action in critical moments. It became a deliberate instrument to attack and discredit scientists and health professionals for political gains. While WHO encourages responsible communication and flags misleading content, social media such as Meta has decided to drop fact checking (Anonymous Lancet 2025a). Deliberate disinformation on both classical and social media is extensively and increasingly used by authoritarian regimes to subvert liberal democracies (Applebaum 2024). To this attack comes now the downsizing of the very institutions we need for fighting the spread of epidemics by the current Trump administration (Anonymous Lancet 2025b, 2025c). The US withdrawal from WHO, the dismantling of USAID, the mass lay‐offs at CDC, the freezing of research money at NIH, and the intention of the new US health secretary to instruct the NIH to take ‘a break’ from infectious diseases, all these measures are serious blows for pandemic preparedness. While one might still understand the unwillingness of US taxpayers to support fragile and underfunded health systems run by African governments, which in addition foster epidemic spread by wars (Wayengera 2024), such political decisions in affluent societies are shortsighted since epidemics do not know frontiers. Even large oceans are no physical barriers to epidemic spread as dramatically seen for West Nile Virus epidemic (Brüssow and Figuerola 2025), COVID‐19, the 2022/3 mpox epidemic and the avian influenza epidemic, which all had major impacts in the US. Sadly, the current US government follows chaotic decisions even when the interests of US citizens are at stake, as in the avian flu/dairy cattle epidemic ongoing in the US (Brüssow 2024). USDA must now try to rehire scientists for an avian flu response who were just fired to cut governmental costs. Viral epidemics are increasing in frequency. This is now the worst moment to upset public health institutions such as CDC, which were the envy of the world. CDC websites were taken down and a prime source of public health information such as CDC's MMWR failed to appear for political pressure, which has not happened in 60 years. Scientists and doctors must stem this erosion of public health and infectious diseases research in the US. The US government deliberately renounces being the leader of science in general and in infectious diseases research, in particular. European countries must try to fill this void created by the current US administration. The European countries have now realised that they must stand on their own feet and spend hundreds of billions of euros on defence. Scientists in Europe should lobby that defence does not only mean to withstand the military threat of dictatorial systems but that defence should also include protection of their populations from future pandemic threats. If a fraction of the planned defence budgets is taken to fight infectious diseases, a lot can be done (e.g., a build‐up of the European CDC) and would also facilitate acceptance of the huge defence budgets by European peaceniks as well as stimulate the soft power of the EU.

## Author Contributions

**Harald Brüssow:** conceptualization, investigation, writing – original draft.

## Conflicts of Interest

The author declares no conflicts of interest.

## Data Availability

The author has nothing to report.
